# A qualitative study of the knowledge, attitudes, and behaviors of people exposed to diesel exhaust at the workplace in British Columbia, Canada

**DOI:** 10.1371/journal.pone.0182890

**Published:** 2017-08-25

**Authors:** Mandy Pui, Anne-Marie Nicol, Michael Brauer, Farshad Palad, Christopher Carlsten

**Affiliations:** 1 Department of Medicine, University of British Columbia, Vancouver, British Columbia, Canada; 2 Faculty of Health Sciences, Simon Fraser University, Vancouver, British Columbia, Canada; 3 School of Population and Public Health, University of British Columbia, Vancouver, British Columbia, Canada; Telethon Institute for Child Health Research, AUSTRALIA

## Abstract

**Purpose:**

To identify exposure-related knowledge, attitudes and behaviors of individuals occupationally exposed to diesel exhaust (DE); to reveal strengths, knowledge gaps and misperceptions therein.

**Methods:**

A Mental Models approach was used to gather information about current scientific understanding of DE exposure hazards and the ways in which exposure can be reduced. Thirty individuals in British Columbia who were regularly exposed to occupational DE were interviewed. The audio was recorded and transcribed. Data was grouped together and examined to draw out themes around DE awareness, hazard assessment and risk reduction behaviors. These themes were then compared and contrasted with existing grey and research literature in order to reveal strengths, gaps and misperceptions regarding DE exposure.

**Results:**

Study participants were aware and concerned about their exposure to DE but had incomplete and sometimes incorrect understanding of exposure pathways, health effects, and effective strategies to reduce their exposures. The perceived likelihood of exposure to DE was significantly greater compared to that of other work hazards (p<0.01), whereas the difference for their perceived severity of consequences was not significant. There was no universally perceived main source of information regarding DE, and participants generally distrusted sources of information based on their past experience with the source. Most of the actions that were taken to address DE exposure fell into the area of administrative controls such as being aware of sources of DE and avoiding these sources.

**Conclusions:**

This study of the knowledge, attitude, and behavior of those occupationally exposed to DE found, most notably, that more education and training and the creation of a health effects inventory regarding DE exposure were desired.

## Introduction

### Occupational exposure of DE

The International Agency for Research on Cancer (IARC), part of the World Health Organization, reclassified DE (DE) as Group 1 (*carcinogenic to humans*) in June of 2012 [1]. DE exposure is also associated with cardiovascular disease, neurophysiological and respiratory symptoms, and consequent premature death in several studies [2–4].

DE is the end product of the combustion of diesel fuel, which is a complex mix of gases and particles. DE is a very common workplace hazard because of the widespread use of diesel fuel and engines. CAREX Canada (CARcinogen EXposure, a multi-institution research project that is generating an evidence-based carcinogen surveillance program for Canada) estimates that approximately 897,000 people in Canada are exposed to some level of DE at the workplace [5]. Specifically, approximately 108,000 people are exposed in British Columbia, making it the second most common workplace carcinogen (after solar radiation) [5]. The Occupational Cancer Research Centre conducted a study to estimate the burden of cancer attributable to occupational DE exposure in Canada from 1961 to 2001 and found that 1.4 million people were occupationally exposed to DE during the 40-year period, and the attributable fraction of lung cancers due to occupational DE exposure was 2.7% [6]. To put it into perspective, with 26,600 Canadians diagnosed with lung cancer annually, 718 cases of those are attributable to occupational DE exposure [7]. Those at particular risk for high exposures include miners, loggers, heavy equipment operators, trucking company workers, and forklift operators in the mining, forestry and construction industries which are very prominent in British Columbia [8].

### Health and safety agencies

Health and safety agencies such as WorkSafeBC, the Canadian Centre for Occupational Health and Safety (CCOHS), the National Institute for Occupational Safety and Health (NIOSH), and the Occupational Safety and Health Administration (OSHA) provide information about DE, its health effects, and possible mitigation strategies. Given the limitations of the study budget and for the purposes of direct comparison, the information readily available on the websites of the health and safety agencies are shown in Table 1.

**Table 1:** Information from the websites of health and safety agencies.

Health and Safety Agency	Acronym for DE	Education	Recommendations	Exposure Limits	References
WorkSafeBC	“diesel fuel, as total hydrocarbons, inhalable”	Smoke colour indicators Exposure route	Servicing engines according to manufacturer Using more than CO as exposure indicator	8-hour time weighted average limit = 100mg/m^3^	[9]
CCOHS	DE	Health effects and mitigation strategies IARC re-classification Types of occupations at high risk	Use reformulated diesel or biodiesel Low-emission engines Exhaust extractor hoses for idling vehicles Respirator if other methods not effective or suitable	N/A	[10]
NIOSH	DE	DE Miners Study results Underground miners 10x exposure as in other workplaces Health effects	N/A	N/A	[11]
OSHA	Diesel particulate matter	Health effects and mitigation strategies IARC re-classification Types of occupations at high risk	Use filters, oxidation catalysts Cleaner-burning engines Restrict amount of diesel-powered equipment in an area to not exceed ventilation capacity	Underground miner 8-hour time weighted average limit = 160μg/m^3^	[12]

### Basic responsibilities and rights for workplace health and safety

Even though parties other than the employee such as the government, employer, manager or supervisor, and health and safety committee (if applicable) are responsible for the employee’s health and safety at the workplace, there are a few reasons that it is important for the employees to be aware of the health effects of DE exposure, and measures to prevent or minimize DE exposure at the workplace [13]. First, it is the employees’ right to be informed about and to know about actual and potential dangers at the workplace. Second, if they are informed about actual and potential dangers in the workplace, then they have a responsibility to use PPE and other measures to prevent and minimize exposure as directed by the employer. Furthermore, they are able to recognize when work is unsafe and proceed to report workplace hazards and dangers as it is their responsibility to do so, or to refuse unsafe work when necessary, as it is their right to do so.

### Workers’ knowledge, attitudes, and behaviors

The purpose of this study is to understand what the individual knows regarding their occupational exposure of DE. Furthermore, we are interested in understanding their attitudes and behaviors, along with just their awareness of DE exposure. This additional information is sought because an individual having knowledge of a health risk does not mean that they necessarily believe themselves to be at risk or that they are likely to do anything to prevent themselves from being in harm’s way or apply their rights. Factors in risk reduction have been well studied in public health, demonstration for example the knowledge of the health risk of smoking, driving under the influence and over-eating are not enough to drive individuals to believe they are at risk or to take actions to reduce these exposures [14–16].

A knowledge, attitude, and behavior (KAB) study collects data from a specific population on what they know, believe, or are doing in regards to a specific topic [17]. The study is usually conducted orally using a semi-structured questionnaire and the data can be qualitative or quantitative. KAB studies ‘identify knowledge gaps, cultural beliefs, or behavioral patterns that may facilitate understanding and action’, and also collect evidence for ‘planning, refining, and evaluating advocacy, communication and social mobilization work’.

Few studies have determined workplace knowledge, attitudes and behaviors regarding occupational exposure of DE. The Workplace Awareness and Knowledge Study from the University of British Columbia aimed to explore individuals’ knowledge and understanding of their exposure to various occupational carcinogens including DE [18]. Preliminary findings identified knowledge gaps of potentially harmful workplace exposures and that awareness differed substantially across different exposures. However, the study was not focused on DE and no data specific to DE has been disclosed. No other studies relevant to workplace knowledge, attitude and behaviour regarding occupational exposure to DE have been found.

While the understanding of occupational exposure is important for all exposed individuals, the recent acknowledgment of DE as being *carcinogenic to humans* (IARC Group 1) has increased the urgency for improved health and safety around DE. Recognizing that simply providing information is not sufficient for behavior change, this study set out to gather information on the knowledge, attitudes and behaviours of workers in occupations where DE exposure was occurring. This information was analyzed to examine strengths, gaps, and misconceptions, along with the societal and organizational factors that could influence how new information about DE could be better translated in an occupational context.

### Study design

One effective way of eliciting knowledge, attitudes and behaviors around risks is by using the Mental Models approach [19]. The rationale is that individuals’ beliefs, decisions and behaviors play a critical role in determining their probability and severity of injury from risk, such as DE occupational exposure. In the Mental Models methodology, the beliefs from domain experts and from the relevant homogenous population are elicited and then compared. The domain experts’ beliefs are established in some studies by interviewing experts in the domain with questions similar to those asked from the relevant population. For example, Wood *et al*. interviewed US Army Corps of Engineers with similar questions asked of laypersons for a study on flood preparedness [20]. In other studies, the domain experts’ beliefs are established with a review of relevant literature to identify important factors as well as relationships among them in the system. For example, in order to identify information needs about wildland fires, Zaksek *et al*. established experts’ beliefs by performing an extensive literature review on existing processes of wildland fires [21]. In this study, the latter method (a review of relevant grey and research literature) was used.

## Methods

### Ethics statement

All study participants gave their written informed consent to participate in this study. The study protocol was approved by the ethics committee of the University of British Columbia, Vancouver, certificate number H12-02135.

### Qualitative interview study

**Fig 1:**
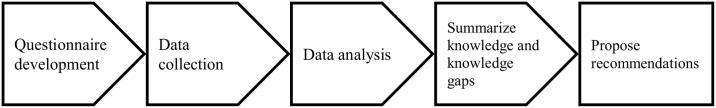
Overall study flow.

This was a qualitative interview study of individuals occupationally exposed to DE conducted according to guidelines for qualitative research [22]. The overall study flow is shown in Fig 1.

### Sample

Subjects were recruited by local advertising (by posting flyers in several locations thought to be likely to be frequented by those exposed to DE, such as a Starbucks coffee café next to a construction site, as well as posting advertisements on the local Craigslist website). Subjects were also recruited by word of mouth, and through contacting unions that were believed to have members who were regularly exposed to DE. To be eligible for the study, the study participant needed to be working in a position where they were regularly exposed to DE at their workplace. They also needed to be older than 19 years old, and have worked for at least one year at their current workplace. Eligible, interested and willing subjects were given an informed consent form and interviewed on a first-come, first-recruited basis. It was decided that participation was limited to four subjects per workplace and six per occupation to prevent narrowing the occupational focus. A heterogenous sample of 30 study participants was recruited between January 2013 and February 2014. No subject who submitted the informed consent form dropped out of the study.

### Interviews

The interview questionnaire was designed such that the data would be collected using the Mental Models methodology. Given the recent attention paid to DE and the limitations of the study budget, expert elicitation was not undertaken with interviewing experts. Instead, the questionnaire was developed by a collaboration between the primary author and three domain experts. The domain experts also provided suggestions on relevant grey and research literature to review and reference for the questionnaire. Examples of grey literature included the websites of WorkSafeBC, CCOHS, NIOSH, and OSHA as well as web links of ongoing studies. Research literature included ones found through the PubMed search engine that were related to occupational exposure, occupational DE exposure, or DE health effects. The questionnaire underwent iterative evaluation with the domain experts, as well as a librarian with expertise in qualitative research. See S1 Appendix for the study questionnaire.

The study questionnaire contained basic questions to establish demographics. The study subjects were asked to report in integers their age and the number of years that they have worked where they were occupationally exposed to DE. They were also asked if they currently or previously smoked to gain a general understanding of how concerned they were about their health. They were also asked if they had lung conditions or occupational injuries and diseases, as that might increase their knowledge level when they were personally affected.

The questionnaire contained questions about the nature of their work as well as their knowledge, attitudes and behaviors regarding the occupational exposure of DE. Some questions were open-ended, for example, “What do you think would happen to a person as a result of DE exposure?” Some questions required the subjects to provide a list of responses, such as to list the top five perceived hazards at work, or to list any recommendations that they could think of to better address DE exposure. Additionally, some more-structured questions were asked to obtain quantitative answers, such as “On a scale of one to five, how much DE do you think you are exposed to at work?” and “What is the actual level of DE that you are exposed to at work?” Some of the questions were followed up by probes designed to elicit more information on a particular subject matter, such as “Can you give me an example?” or “When might this occur?”

Throughout the study, one interview question was changed. After the first 15 subjects were interviewed, the domain experts suggested that it would be helpful to ask the subjects, in addition to their perceived level of occupational DE exposure, if they knew the actual level of DE that they were exposed to at work. Thereafter, the remaining 15 subjects were asked “*what is the actual level of DE at your workplace*?”

The study involved one-on-one interviews conducted by the same interviewer (M. Pui) to maintain consistency in the style of the questioning. The interviews were conducted between the interviewer and the study participant with no other individuals present, at a private and quiet location, which were usually in-person at the interviewer’s worksite, or over the phone. The average time for these interviews was 30 minutes. The interviews were audio-taped, and the interviewer took notes.

### Data analysis

The audio recording of the interviews was transcribed using the NVivo software. The Mental Models approach was used for the initial coding schema for data analysis; like the development of the questionnaire, it was informed by a review of grey and research literature to develop model categories. An example of the development of the coding schema is as follows: Nicol and Hurrell conducted a similar study to determine the knowledge, attitude and behavior of individuals who were occupationally exposed to metal working fluids, a dermal and respiratory irritant [23]. Informed by this research literature, the categories of “sources of information for a work hazard” and “trusted sources of information” were added to the initial coding schema.

In this sense, the initial coding schema acted as the template for the expert understanding of DE, its health effects and the strategies that could be used to reduce exposure. Then the respondents’ responses were read, re-read, and examined to identify additional categories and properties, to infer interrelationships, and to generate a theory. An example question would be “*Can you tell me what you think would happen to a person as a result of DE exposure*?*”* The known health effects that result from DE exposure (eg. lung cancer, asthma) were taken from the literature. However, if an individual’s responses did not fit into the initial coding schema, then additional codes were created (eg. if the response was “problems with lungs”). This allowed for both well-known answers along with participants’ less specific or incomplete understanding of exposure. If survey responses deviated completely (eg. an impossible health effect), the result was included to ensure completeness of respondents’ perceptions of exposure.

Responses for each code were grouped together and counted. A representative quote for each group of responses was shown to illustrate themes, while making a concerted effort to include anything that was relevant to DE. In the quotes shown, some sections were edited for the purpose of maintaining the subjects’ anonymity, or to make the quotes more concise, or to correct the grammar. The categories and themes elicited from the study participants were compared and contrasted to those established from the expert beliefs from the research literature, and illustrated and discussed in the Discussion section. In the case of quantitative answers from the more structured questions, such as those regarding demographics, and ranking responses from “on a scale of one to five…” questions, results were counted up, put in categories if possible, compared by Wilcoxon rank test if applicable, and shown in tables and figures. If applicable, quotes were collected, and a representative quote for each group of responses was shown.

## Results

### Participants

Thirty eligible participants were successfully recruited into the project over a one-year period. The characteristics of the 30 study participants are outlined in Table 2.

**Table 2:** Participant characteristics.

**Characteristic**	**Average (SD)**
Age	46.3 (13.6)
Years Exposed to Occupational DE	20.0 (12.8)
**Characteristic**	**N**
Male	25 (83%)
Smoker	2 (7%)*
Previous Smoker	7 (25%)*
Known Asthma	6 (21%)*
Known Allergies	10 (36%)*
Other Lung Conditions	1 (3%)*
Occupational Disease or Injury	3 (11%)*

* Data for this variable was only obtained for 28 subjects

The subjects’ ages ranged from 22 to 69. The duration that the subjects were occupationally exposed to DE ranged from one year to 50 years. The cohort of study subjects consisted mostly of male non-smokers. Some of the data was only obtained for 28 or 29 subjects because two subjects refused to answer some of the questions. Those questions will be noted as such in the Results and Discussion sections.

One subject reported to have a lung condition (chronic obstructive pulmonary disease). Three subjects reported to have an occupational disease or injury (chronic pain from a car accident, disc problems, and chronic obstructive pulmonary disease).

### Job titles

The study subjects were asked to give their job titles as Table 3 shows. While the job titles were given by the study participants, the corresponding job sectors were determined with guidance from the Government of Canada Job Bank [24]. Out of 30 subjects, 21 subjects worked in the Transportation sector, while four subjects worked in Rescue Service. One subject worked in the Fishing sector. Three subjects worked in the Construction sector, and one subject worked in Health Sciences.

**Table 3:** Job titles and sectors.

Job Title	Job Sector (Subsector)	# Subjects with Job Titles
Transit Operator	Transportation (Buses)	5
Bus Service Clerk	Transportation (Buses)	2
Vehicle Inspector	Transportation (Cars)	1
Locomotive Engineers	Transportation (Railway)	3
Heavy Duty Mechanic	Transportation (Railway)	1
Airport Crew Member	Transportation (Airplanes)	1
Marine Oiler	Transportation (Ferries)	1
Ferry Engineer	Transportation (Ferries)	6
Fuel Dock Supervisor	Transportation (Boats)	1
Firefighter	Rescue Service	3
Fire Battalion Chief	Rescue Service	1
Commercial Fisherman	Fishing	1
Heavy Equipment Operator	Construction	2
Welder	Construction	1
Lab Technician	Health Sciences	1

The subjects were asked if they have received any safety training at work. If so, they were asked to describe any training that was relevant to DE. Out of 28 subjects who responded to this question, nine subjects reported not having received any safety training at work. Three subjects reported receiving safety training on the job, with two of the three subjects specifying that their training entailed reading safety bulletins “*such as from WorkSafeBC*”. Sixteen subjects reported having done some kind of safety course. Additionally, four of the subjects disclosed that they were part of the health and safety committee at their workplace, and have been accordingly trained for that role. Out of all 28 subjects, all subjects reported that they have not had any safety training that was specific to DE. S2 Appendix (A) shows some of their responses.

### Actual and perceived DE exposure

As stated in the study method, the question about actual DE exposure was changed during the study. Therefore only 15 out of the 30 subjects were asked the question “*What is the actual level of DE that you are exposed to at work*?” From this, 14 out of the 15 subjects reported not knowing. The remaining subject had a very specific answer,

“27.68 parts per million in a volume of [a workplace] that was 50 x 60 feet with a 16 foot ceiling.”

Then he went on to say,

“*We have no clue what [substance] that parts per million is*, *at what stage*, *was that just after starting the truck*, *or when you’ve worked in it all day*, *or after letting it smoke out*. *You don’t know how much of it is going through your gas mask if you wore one*.*”*

**Fig 2:**
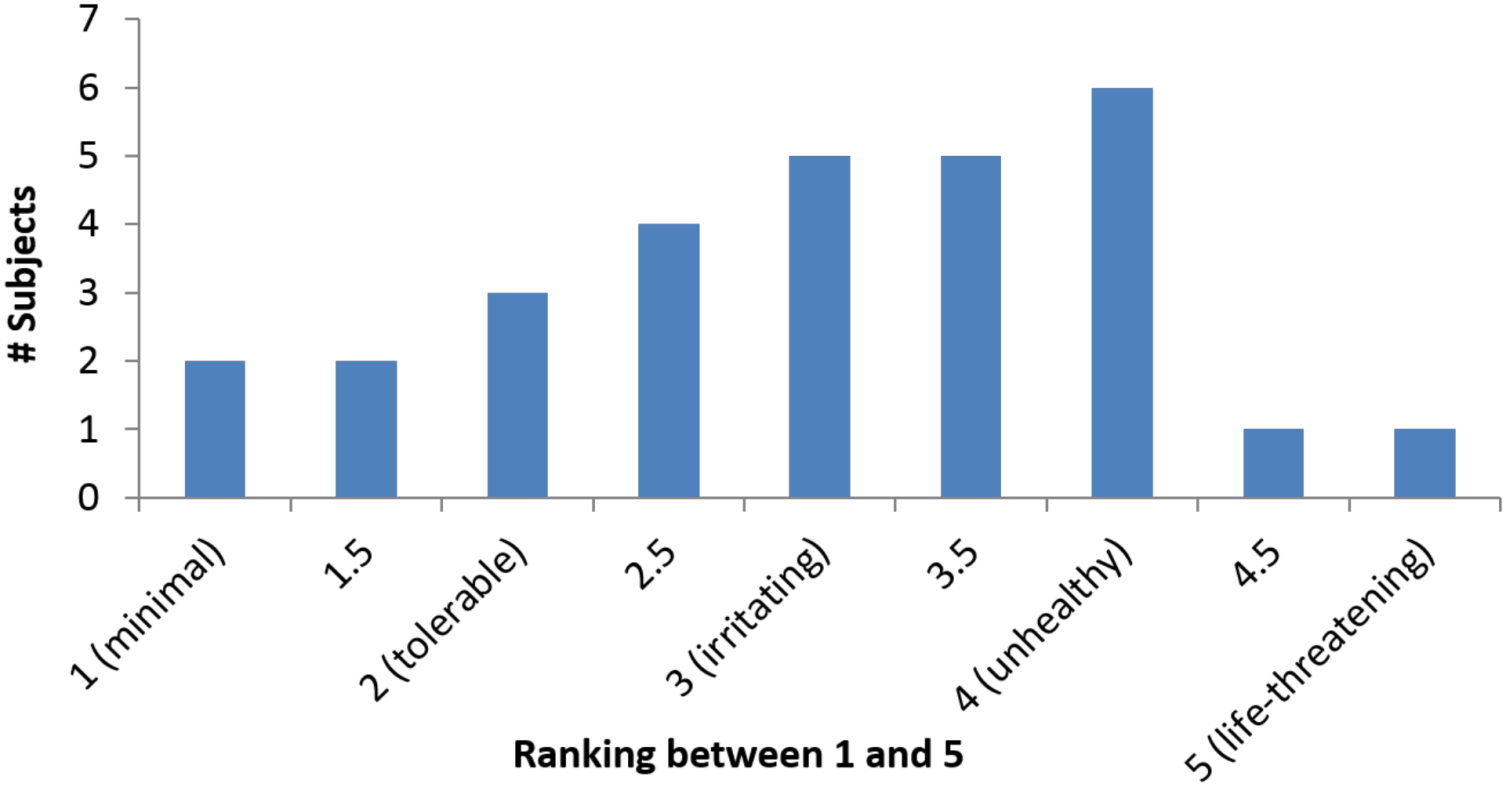
Subjects’ perceived exposure level of DE.

Since most of the subjects were not given information regarding the actual DE exposure, the study subjects were also asked about their perceived DE exposure: *“On a scale of one to five*, *how much DE do you think you are exposed to at work*, *one being minimal*, *two being tolerable*, *three being irritating*, *four being unhealthy and five being life-threatening*?*”* Twenty-nine subjects answered, as Fig 2 shows.

### DE and other work hazards

The subjects were asked *“what are top five hazards at your workplace*, *if you could name any hazards*?*”* Out of 29 subjects who responded, 19 (66%) included DE as one of their top five perceived hazards, while the other 10 subjects did not.

Table 4 shows the top five perceived occupational hazards in categories determined with guidance from a list of hazards from Occupational Safety and Health Administration [25].

**Table 4:** Subjects’ perceived top occupational hazards.

Hazard Categories	Top Perceived Hazards Listed by Subjects (Frequency)
Safety	Motor Vehicles (14), Slips Trips and Falls (13), Physical Injury (10), Fire (4), Electrical Hazards (4), Machinery Failure (4), Weight of Diesel Engine (3), Explosions (3)
Biological	Pathogens (3), Viruses (3), Toxins (2), “Needles” (2)
Physical	Aggressive People (7), Drowning (3), Noise (3), Vibration (2), Dehydration (2)
Ergonomic	N/A
Chemical	DE (19), Asbestos (4), Fiberglass (4), Fire Smoke (3), Paint Enamel (3), Other Fumes (3), Solvents (2), Cleaning Agents (2), Welding Exhaust (2), Chemical Hazards (2), Handling of Fuel Systems (2)
Work Organization	Stress (6), Fatigue or Sleep Deprivation (4), Shiftwork (2), Workplace Violence (2), Unhealthy Lifestyle (2), Cardiac Issues (1)

After the subjects named their top five perceived hazards, they were asked to put each of their named hazards on a scale of one to five, in terms of likelihood that the hazard would occur at the workplace, with one being “will not happen”, two being “might happen”, three being “likely to happen”, four being “very likely to happen”, and five being “sure to happen”. Then, they were asked to put each of the named hazards on a scale of one to five in terms of severity of the consequences if the hazard was to occur, with one being “normal”, two being “mild”, three being “moderate”, four being “severe”, and five being “extreme”.

The ranking results for DE *vs*. other named hazards were compared, using only the responses provided by the 19 subjects that included DE as one of their top five perceived hazards. The other hazards listed by each subject were grouped together and the average was obtained. Regarding the subjects’ perceived likelihood of the hazard occurring at the workplace, the average was 4.0 for DE, and 3.2 for the other hazards. According to a Wilcoxon signed-rank t-test, the difference was statistically significant (p<0.01). Regarding the subjects’ perceived severity of consequences if the hazards were to occur, the average value was 3.7 for DE, and 3.2 for other hazards (p>0.05).

Out of the other hazards named by the workers, 12 were inhalational hazards. These were *“pathogens”*, *“toxins”*, *“viruses”*, *“fire smoke”*, *“chemical hazards”*, *“paint enamel”*, *“solvents”*, *“cleaning agents”*, *“welding exhaust”*, *“asbestos”*, *“fiberglass”*, and *“fumes”*.

**Fig 3:**
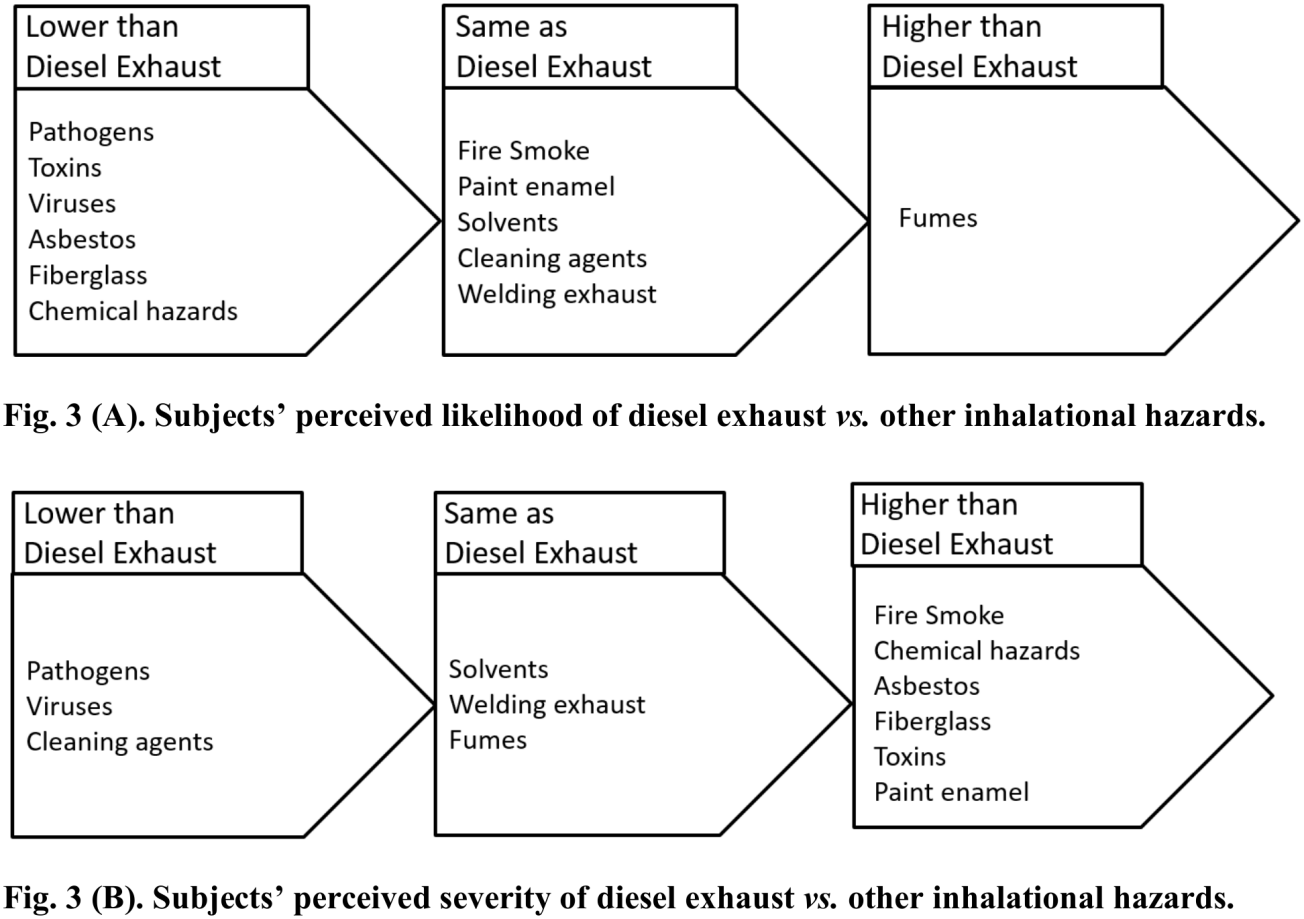
Subjects’ perceived likelihood and severity of consequences due to DE *vs*. other inhalational hazards.

The inhalational hazards that were ranked lower in likelihood as compared to DE were *“pathogens”*, *“toxins”*, *“viruses”*, *“asbestos”*, *“fiberglass” and “chemical hazards”*, as Fig 3A shows. *“Fire smoke”*, *“paint enamel”*, *“solvents”*, *“cleaning agents”* and *“welding exhaust”* were ranked the same as DE in likelihood, while *“fumes”* were ranked to be more likely to occur than DE. The inhalational hazards that were ranked lower in severity by comparison to DE were *“pathogens”*, *“viruses”*, and *“cleaning agents”* as Fig 3B shows. *“Solvents”*, *“welding exhaust”* and *“fumes”* were ranked to be as severe as DE. Meanwhile, *“fire smoke”*, *“chemical hazards”*, *“asbestos”*, *“fiberglass”*, *“toxins”*, and *“paint enamel”* were perceived to be more severe than DE.

The reasoning for the rankings of DE and other inhalation hazards seemed to be subjective and based on personal experience. See S2 Appendix (B) for representative responses.

### Health effects of DE

The subjects were asked *“Can you tell me what you think would happen to a person as a result of DE exposure*?*”* They were asked to name as many health effects as they could. Out of 30 subjects who answered this question, 21 subjects (70%) mentioned some form of respiratory issue, 17 subjects (57%) mentioned cancer, three subjects (10%) mentioned headaches, three subjects (10%) mentioned throat irritation, two subjects (7%) mentioned eye irritation, two subjects (7%) mentioned circulatory system issues, and two subjects (7%) mentioned nervous system issues. One person who mentioned cancer specified that it would cause leukemia while another subject specified that it would occur “*only in their late 60’s or 70’s*”. There was one response for each of the following: nausea, throat deterioration, birth defects, allergy to diesel, multiple sclerosis, and toxicity to body. Four out of 30 subjects (13%) said that they did not know of any health effects and did not name any health effects.

Regardless of whether the subject mentioned cancer or not, each subject was asked later in the interview, *“do you think DE is a carcinogen*, *meaning something that causes cancer*?*”* Out of 30 subjects, 23 subjects (77%) said yes, five subjects (17%) said that they did not know, and two subjects (7%) said no. Regardless of whether the subject agreed or disagreed to DE being a carcinogen, or admitted that they did not know, it seemed that none of the subjects had any evidence to support their opinion. See S2 Appendix (C) for representative responses.

### Trusted sources of information

Thirty subjects responded about the sources of information that they could recall regarding diesel exhaust, and the sources of information that they would trust, as Table 5 shows. See S2 Appendix (D) for representative responses.

**Table 5:** Subjects’ sources of information.

Sources of Information	# Subjects that Mentioned Source	% (within the *# Subjects that Mentioned Source*) that Trust Source
WorkSafeBC	22	59
Internet	21	90
Doctors	14	86
Employer	14	36
Unions	10	30
Health Canada	9	100
Health and safety committee	7	29
Universities and research studies	5	100
Libraries	5	100
Colleagues	4	50
Government	4	50
NIOSH	3	100
BC Cancer Research Centre	2	100
US Environmental Protection Agency	2	100
Newspapers	2	100
Firefighter associations	2	100
Information recommended by media	2	100
PubMed	1	100
National journals and reference materials	1	100
Centers for Disease Control and Prevention	1	100
OSHA	1	100
Coastal Health	1	100
Ministry of Health	1	100
Health authorities	1	100
National Resources Defense Council	1	100
Green Fleets	1	100
Canadian Auto Workers	1	100
BC Medical Association	1	100
BC Lung Association	1	100
Environment Canada	1	100
Mayo Institute	1	100
Government-issued American studies	1	100
Television	1	100
Private agencies	1	0

### Actions to address DE

The subjects were asked about all the actions that they took at their workplace to address DE exposure and were asked to name as many as they could. Their responses here are organized by the different methods of hazard control: personal protective equipment, administrative controls, and engineering controls. See S2 Appendix (E) for representative responses.

Five out of 30 subjects mentioned using personal protective equipment to address the DE exposure, as in using a mask with either a filter or a cartridge. Only one of the five subjects said that she and her coworkers wore the masks regularly. The other four mentioned using a mask with a few caveats.

The subjects mentioned addressing DE by an administrative control, meaning a control that altered the way the work was done. Specifically, 14 out of 30 workers mentioned being aware of sources of DE and avoiding these sources, which one of the subjects called “*situational awareness*” and described it as being aware of “*the weather conditions*, *which direction the wind is blowing*, *trying not to be downwind from the exhaust”* while one of the other subjects talked about avoiding DE but with the caveat of hurrying parts of the job. Six subjects mentioned holding their breath as a strategy to address the exposure of DE. One subject believed that this action was sufficient, as “*[the exposure] only take a few seconds*, *right*?*”* Two subjects filled out exposure or incident forms. However, the two subjects had different thoughts on how best to keep records of occupational exposure: one advocated for keeping a record of all the exposures that one has throughout a career, while the other subject thought that it was not a useful system as he would realistically have to fill out paperwork every day which he did not do. Two subjects used a monitor or a carbon monoxide tester. Four subjects mentioned fixing leaks as soon as possible to minimize the amount of DE in the air. Two subjects changed the timing and type of shift so that they were exposed to lesser levels of DE. Four subjects mentioned idling less; one of the subjects was involved with a company-wide campaign to reduce idling. Two subjects attended tailgate safety meetings to discuss with fellow coworkers about the exposure of DE. Each of the following actions was mentioned by one out of 30 subjects: Using better grade diesel, sending in maintenance requests, being familiar with equipment, talking to his manager, and cleaning soot from pipes.

Twenty-two of the 30 subjects mentioned addressing DE by an engineering control, which included modifying equipment, systems, and processes that reduced the source of exposure. Three subjects mentioned using a more efficient engine with newer technology. Nineteen subjects mentioned addressing ventilation and many different methods were mentioned, to either increase airflow that introduced fresh air into an area, or to redirect or decrease airflow that included DE

### Recommendations to better address the exposure of DE

The subjects were asked to share their recommendations for how to better address the exposure of DE at their workplace, whether it was by themselves, fellow workers, the workplace, the government, etc. They were asked to name as many recommendations as they could think of. See S2 Appendix (F) for representative responses. The workers wanted the workplace to provide adequate protective equipment and appropriate training to use the equipment. The workers recommended for workplaces to formally recognize DE as a hazard, and to offer education and training relevant to DE, regular testing for the workers’ health as well as for the exposure, and regulations as appropriate for such an exposure. In addition, the subjects suggested creating an inventory of health effects. Furthermore, subjects recommended changes in the diesel fuel or the engine, equipment maintenance, facility improvements, as well as changes in the workplace that increased ventilation and cut down on emissions

## Discussion

### Domain experts’ beliefs

As mentioned, the domain experts’ beliefs informed the study questionnaire and the initial coding schema for data analysis. The experts’ beliefs in this study were established by a review of relevant grey and research literature as suggested by three domain experts, and an example was websites of WorkSafeBC, CCOHS, NIOSH, and OSHA. At the outset, it was expected that best practices were established by and easily accessible from health and safety agencies. A study limitation was that the websites of the four health and safety agencies that attributed to the domain experts’ beliefs did not provide consistent, comprehensive and up-to-date information. For example, each of the websites should use the ALARA “as low as reasonably achievable” principle since DE was reclassified as *carcinogenic* (Group 1) in 2012 but none did. DE should be consistently named as DE since that was how the study participants referred to it as well. If the DE was referred as “diesel particulate matter” or “diesel fuel, as total hydrocarbons, inhalable”, it might be misinterpreted as another substance. In the future, it may yield more accurate results if the domain experts’ beliefs are elicited by interviewing the experts in the domain with questions similar to those asked from the individuals exposed to DE.

### Participant characteristics

As expected based on the job sectors from which participants were recruited, 83% of the subjects were male. For example, 75% in the transportation industry are male, while 88% of the people in the construction industry are male [26]. Overall, 53% of the labour force in British Columbia are male [27].

### Job titles

The job titles and job sectors represented in this study were expected from subjects working in British Columbia. However, some of the largest exposed groups were not represented, such as truck drivers, subway drivers, couriers, and taxi drivers, despite efforts to recruit them for this study [28].

### Safety training

When 28 subjects responded about safety training, nine of them (32%) reported not having any training. None of the subjects believed that they had any training relevant to DE.

Three subjects mentioned that their safety training was obtained through working on the job instead of through formal training. Another study found it typical for employees to learn from one another and to draw on more experienced individuals as a source of knowledge, rather than from written information or through formal training [29]. This informal training might confer dangerous practices, as well as training inconsistency and undue dependency on a given employee’s colleagues. WorkSafeBC started to enforce orientation and training for new workers in 2007, which included training specific to the workplace and the hazards, and training to non-new workers on new processes and equipment [30]. However, upon review of online resources from several health and safety agencies, there were no explicitly-stated requirements for workplaces to provide DE-specific education or training to employees. WorkSafeBC and other health and safety agencies needed to understand workers’ culture of learning through one another while putting more emphasis on checking employer due diligence and enforcing regulations and accountability on DE-specific education and training.

### Perceived and actual DE exposure

All of the subjects were at least aware of the exposure of DE at work. The majority of the subjects (18 out of 29 subjects) were at least bothered by the exposure and perceived their level of exposure to be irritating, unhealthy, or life-threatening.

The subjects’ opinions about the exposure of DE were in stark contrast to their knowledge of the exposure. Fourteen out of fifteen subjects that were asked about actual DE exposure did not know the level that they were exposed to. Upon a review of the transcripts, there was a general lack of knowledge about the individuals’ level of occupational exposure as well as their right to know about their exposure. This identifies a potential lack of education being provided by the employers of these workers.

### DE perceived as a hazard

The majority of the study cohort (19 out of 29) mentioned DE as one of their top five perceived occupational hazards. Furthermore, subjects’ perceived likelihood of DE was significantly higher than other hazards, whereas subjects’ perceived severity of consequences due to DE compared to other hazards was not significantly different. A limitation of this study was that open-ended questions were asked of the subjects, which resulted in many of the answers given for the inhalational hazards not specific enough to associate with a particular hazardous material.

### Health effects of DE

The benefits to workers occupationally exposed to DE having a more complete and thorough understanding of the associated health effects is worth noting. Researchers have found that a lack of awareness of health effects on the part of the exposed individuals made the correct diagnosis of occupational disease very challenging and that this would likely lead to delays in reporting and under-reporting of diseases [31, 32]. Ultimately, individuals exposed to DE need to know and understand the health effects of DE so that they are more likely to report accurately to their doctors with a valid claim, and perhaps more likely to get compensation through WorkSafeBC and Workers’ Compensation Board.

Respiratory issues, cancer, circulatory system issues and nervous system issues are established health consequences caused by the exposure of DE. However, many subjects were not able to recall these health issues: nine subjects (30%) with respiratory issues, 13 subjects (43%) with cancer, 28 subjects (93%) with circulatory system issues, and 28 subjects (93%) with nervous system issues. One possible explanation for there being so many more individuals not being aware of circulatory and nervous system issues than with respiratory issues and cancer might be because research on these health effects was more recent than research on respiratory effects and cancer associated with DE. For cognitive issues in particular, attention has been scarce until recently, whereas research on respiratory effects and cancer was done as early as the 1980s [33–37]. Of particular interest were circulatory issues; a significant body of research has been produced on this issue in recent years [3, 35–43]. The abundance of literature available about this particular health effect was in stark contrast to the lack of knowledge in the subjects surveyed; only two subjects mentioned cardiovascular issues. It is worth emphasizing that more efforts need to be made to communicate the circulatory system issues associated with DE.

A difference between the subjects’ responses and the information from the health and safety agencies and scientific literature was evident in the knowledge of technical terms. For example, it was not clear what the subject meant when he said that DE caused “toxicity to the body”. This was not surprising for a layperson to not have detailed and technical information on diseases that he did not have. As Sadhra *et al*. pointed out, it would be wrong to equate difficulties of articulation with lack of understanding [29].

Subjects’ responses (eg, that DE was associated with birth defects, multiple sclerosis, and leukemia and that cancer would occur “*only in late 60’s or 70’s*”) indicate an incomplete, and sometimes incorrect, understanding of the health effects of DE. It was noteworthy that these responses were all serious health effects, which contrasted with results from Sadhra *et al*. which indicated that individuals tended to mention more about common health problems compared to those that were seen as more serious [29]. Furthermore, this suggested that individuals were not aware of the correct sites of the body that DE could affect. It was concerning that four out of 30 subjects (13%) said that they did not know of any health effects and did not name any health effects.

### Trusted sources of information

Thirty subjects listed a total of 33 different sources of information. Twenty-nine subjects named two or more sources of information. One subject named as many as eight sources. This suggested that there was not one source of information that was universally perceived as the “go-to” source of information about occupational exposure of DE.

The most-mentioned source of information was WorkSafeBC, which was not surprising as the protection agency was perceived as heavily involved in workplace health and safety and would have numerous bulletins and safety signs with their organization name in most of the workplaces in the province. However, it was concerning that nine out of 22 subjects who mentioned WorkSafeBC (41%) would hesitate to trust the agency.

Twenty-one out of 30 subjects mentioned using the internet, which suggested the importance of personal control when it came to informing themselves about DE.

One of the subjects who would trust Health Canada brought up the concern that Health Canada might not have the relevant information. An online search of the Health Canada website revealed that they did in fact have a lot of relevant information, such as information about acute and long-term health effects [44]. However, there was no information about cardiovascular disease or cancer associated with exposure to DE, or information for occupational exposure and actions to take to address occupational exposure.

Twelve out of 14 subjects that identified their doctor as a source of information said that they would trust their doctors. However, it was unclear whether this could be a sustainable and realistic source of information about the exposure of DE to everyone who was occupationally exposed.

Upon review of the feedback from the subjects, it was not surprising that there was no one universally perceived go-to source of information as each source of information had its flaws. This identifies the importance of providing proper onsite education about DE exposure to ensure workers are receiving adequate and accurate information about DE exposure.

### Using personal protective equipment

There were many caveats of using a mask regularly and consistently across all workplaces where individuals were exposed to DE, as mentioned by the subjects. Unless the wearing of masks was a rule implemented by the workplace or the government and everyone who had contact with DE at work were seen to wear masks, the few people who wore the masks would be judged and teased by their colleagues, or the public if they came in contact with the public, which would discourage the wearing of the masks. Also, there was no financial support for masks or respirators. Furthermore, subjects seemed to be more likely to wear protective equipment when they could smell noxious fumes, such as when they were painting. However, with DE, the fumes might not be perceived as noxious as other fumes so the protective equipment could be perceived as not as necessary. Another caveat to wearing masks regularly were instances mentioned by study participants where the wearing of masks or respirators would hinder their job performance or safety. Yet another caveat to wearing masks was the belief that some of the subjects had about DE not having any health effects or only affecting some people.

Subjects recommended for personal protective equipment to be provided along with appropriate training. When masks or respirators were dangerous, cumbersome or impractical to use for certain work duties, an alternative protection method needed to be provided.

### Administrative controls

Most of the actions that workers took to address DE exposure fell into this category. The many responses about being aware of, avoiding, and holding their breath near DE suggested that many individuals’ instinct was to avoid the DE and to control their own dose of DE, even if their actions were in fact not useful for the constant exposure of DE. Also, it was concerning that the individuals might be doing so with the caveat of hurrying parts of their job and therefore putting their job performance or their safety on the line.

A few effective actions were mentioned but only by one to four subjects per action: fixing leaks, idling less, changing the timing and type of shift, using better grade diesel, sending in maintenance requests, being more familiar with equipment, and cleaning soot from pipes. It would be worthwhile to discuss with all individuals exposed to DE about these actions being effective to take at the workplace. The action of one study subject to quit his job was an effective method to remove himself from the exposure of DE. Ideally, individuals were able to keep their jobs and be able to work safely and healthily.

Subjects’ recommendation for the workplace to formally recognize DE as a hazard and to offer education or training about DE and health effects, actual or potential hazards, and appropriate actions to take was most frequently mentioned out of all the recommendations. This was aligned with the subjects’ feedback that none of them have had DE-specific safety training.

The subjects’ recommendations to have regular health check-ups in the context of DE exposure throughout their careers, and to maintain a health effects inventory, were excellent recommendations for several reasons. First, the information that they received during their check-up would be personalized to each individual and so each individual would be better informed and more motivated to protect their own health. Second, there was still more research that could be done about the health effects and effective mitigation strategies associated with DE, and this would be able to provide researchers with more information. Third, this would better inform workers’ compensation boards on the effects associated with occupational exposure to DE.

### Engineering controls

Study subjects mentioned several effective engineering controls to reduce occupational exposure to DE such as redirecting fumes, using a more efficient engine with newer technology, maximizing ventilation, using a fan, filters, and using an extraction system.

However, it was concerning that all of these actions but one (maximizing ventilation) were only mentioned by two to five subjects each (less than 20% of subjects).

It might be worthwhile for individuals to know what engineering controls existed at their workplace so that they were aware of the degree at which they were already protected and how much they needed to take it upon themselves to protect themselves from the exposure of DE.

### Workers without any actions or recommendations

One of the two subjects who said that they did not take any action to address occupational exposure of DE corresponded to one of the four subjects who did not know of any of the health effects associated with DE.

Interestingly, all five subjects who had no recommendations to better address occupational exposure of DE did take at least one action at their workplace and knew of at least one of the health effects associated with DE. It suggested that these subjects were aware of DE and associated health effects, took action to address it, and were genuinely satisfied with their safety and health at work.

### Study strengths and limitations

A strength of this study was that first-hand knowledge, knowledge gaps, and misperceptions from the people exposed to DE were identified. This was an exploratory study where areas of interest were identified for further research to be conducted to better understand the work culture of any of the occupations or work sectors that were mentioned in this study.

A limitation was that all the study participants knew that the study had to do with DE and therefore the study cohort could be more aware or worried about DE than the general population. It was also possible that the knowledge of this study having to do with DE could have affected their answers, such as when they were asked to list the top five perceived hazards at work. However, this made the fact that the subjects had incomplete, and sometimes incorrect understanding of the DE and health effects even more concerning. Another limitation for this study was that there were only 30 subjects in the study. With the study being only in British Columbia, Canada, the information and recommendations might or might not be applicable to other cities and could not be generalized to people exposed to DE at the workplace at large. However, because the aim of a qualitative study is to generate a deeper understanding about an issue, rather than to create generalizable results, this low number of participants does not impact the analysis.

## Conclusions

In conclusion, this was the first study that has evaluated the knowledge, attitudes, and behaviours of individuals exposed to DE after the declaration from IARC about its carcinogenicity. Key findings include:

1. The majority of the study subjects (18 out of 29) perceived their occupational exposure of DE to be irritating, unhealthy or life-threatening.
2. Many of the subjects could not recall significant health effects associated with DE: respiratory issues, cancer, cardiovascular and cognitive issues. More than 10% (Four out of 30) of the subjects could not name any health effects associated with DE.
3. Nine out of 28 study subjects (32%) reported not having any safety training, and none of the subjects reported having training specific to DE.
4. WorkSafeBC was the most-mentioned source of info, but nine out of the 22 participants (41%) that listed this source would hesitate to trust the agency.
5. The most-mentioned action to address DE exposure is being aware of and avoiding sources of DE.
6. Subjects had many safety and practicality issues with using personal protective equipment to address exposure of DE.

Key needs for the future, as recommended by those included in this research project, are as follows:

1. The employer may help the employees by providing the following: information about the actual levels of DE, resources to measure and monitor DE levels, and education and training regarding mitigation strategies, while understanding employees’ culture of learning through one another.
2. The employer should realize that masks are often impractical to use and other methods of exposure reduction must be prioritized.
3. Individuals’ physicians should be aware of their exposure to DE so as to consider related health conditions.
4. The management and government may have more impact in DE exposure reduction at the workplace by updating regulations according to the latest scientific knowledge and then reinforcing them.

## Supporting information

S1 Appendix Study questionnaire. (DOCX)

S2 Appendix Study subjects’ interview answers. (DOCX)
