# Supplementary material for: A qualitative study of the knowledge, attitudes, and behaviors of people exposed to diesel exhaust at the workplace in British Columbia, Canada
Source: PLoS One. 2017 Aug 25;12(8):e0182890. doi: 10.1371/journal.pone.0182890 (PMC5571928; doi:10.1371/journal.pone.0182890)
Supplement: S1 Appendix — (DOCX) [file pone.0182890.s001.docx]

**S1 Appendix. Study Questionnaire**

Interview Script

Name: ________________________________

Date of Interview: ______________________

Interviewer: ___________________________

I will now interview you about your experience with diesel exhaust at your workplace. Please note that this is focused on just the exhaust coming off of diesel engines at your workplace.

The interview will take about 30-45 minutes, and I will be recording the audio of this interview, which will be transcribed and analyzed.

Thank you for your willingness to participate and be interviewed. I would like to remind you that you can choose what to disclose for any of your answers and that you are free to refuse to answer any of the following questions.

I have been studying the health effects of diesel exhaust, and now I am interested in understanding the experience of diesel exhaust exposure at the workplace from your point of view.

1. Do you have any questions before we start? Please refrain from asking questions until the end of the interview as I would like to make sure that I have a good picture of your experience during our allotted time together.

2. Please tell me your age. ________

3. Please tell me what motivated you to participate in this study?

4. Do you smoke? (pack-years)

5. Do you exercise? (type, frequency)

6. What is the highest level of education that you have attained eg. degree, diploma? Did you receive any safety training at work? If so, please describe any training that is relevant to diesel exhaust.

7. Have you been diagnosed with asthma or allergies?

8. Do you have any occupational disease/injury?

9. Do you have any other lung conditions?

10. Please tell me how many years you have been working at this work site? ______

In this industry? ________

11. Please tell me your job title _____________________

12. What is your job like day-to-day?

13. Please describe to me in as much detail as possible your work site.

14. Could you describe in as much detail as possible a situation at your workplace when you feel like you are being exposed to diesel exhaust? What symptoms did you experience?

15a. How much diesel exhaust do you think you are exposed to at work? Scale of 1-5, see below_______

15b. Please specify for the exposure: duration __________________, frequency______________________, number of years_________.

15c. How much diesel exhaust do you think you are exposed to outside of work? On a scale of 1-5, see below______.

15d. What is the actual level of exposure of DE at work?

16. Please tell me the sources of diesel exhaust at work? Outside of work?

17. What are your thoughts about being exposed to diesel exhaust at your workplace?

18. What are your concerns about being exposed to diesel exhaust at your workplace?

19. What are the top 5 hazards at your workplace? For each, scale of 1-5 how likely to happen? Scale of 1-5 for severity?

20. Can you tell me what you think would happen to a person as a result of diesel exhaust exposure?

21. Describe the actions that you take at your workplace to address diesel exhaust exposure.

22. If you were going to learn about the health effects of diesel exhaust exposure, or maybe some of the hazards that you are exposed to at your workplace, where would you go for this sort of information? Please name all the places.

23. Please list all the persons, agencies, or groups that you would trust for information on diesel exhaust and its possible associated health effects.

24. Could you describe in as much detail as possible a situation when you have talked to someone at your workplace about the exposure of diesel exhaust?

25. Could you describe in as much detail as possible a situation when you have talked to someone outside of your workplace about the exposure of diesel exhaust?

26. Have you ever talked to your doctor about being exposed to diesel exhaust at your workplace?

27. Have you heard anything in the media recently regarding diesel exhaust exposure?

28. Do you think diesel exhaust is a carcinogen (something that causes cancer)?

29. Do you have any recommendations on what health researchers, health and safety organizations, or policy makers can do to address diesel exhaust exposure at the workplace? (separate them, and ask them for specific examples they’ve given)

30. Is there anything else that you would want to add before we end the interview?

Thank you for sharing with you your point of view. [Share with interviewee some of the main points that were mentioned.] How did you experience being interviewed about your experience with diesel exhaust exposure at your workplace?

Possible follow up questions or statements to any of the above questions:

1. Pause or repeating significant words of the answer, to invite subject to go on with the description.
2. Do you have a further example of this?
3. In what condition does this occur? How frequent does this occur?
4. Is it correct that when you said… that you meant….?
5. For a scale of how much diesel exhaust exposed to: 1=minimal 2=tolerable 3=irritating 4=unhealthy 5=life-threatening
6. For scale of likely to happen: 1=won’t happen 2=might happen, 3=likely to happen, 4=very likely to happen, 5=sure to happen
7. For scale of severity: 1=normal, 2=mild, 3=moderate, 4=severe, 5=extreme

Notes: acknowledge concerns/questions, talk about time limit, give email/resources later
