# Supplementary material for: A qualitative study of the knowledge, attitudes, and behaviors of people exposed to diesel exhaust at the workplace in British Columbia, Canada
Source: PLoS One. 2017 Aug 25;12(8):e0182890. doi: 10.1371/journal.pone.0182890 (PMC5571928; doi:10.1371/journal.pone.0182890)
Supplement: S2 Appendix — (DOCX) [file pone.0182890.s002.docx]

**S2 Appendix. Study Subjects’ Interview Answers.**

**(A) “Did you receive any safety training at work? If so, please describe any training that is relevant to diesel exhaust.”**

*“[Since I have] a role on the health and safety committee, I have attended, and I will be attending a safety conference, and go to various safety workshops that are for when you take on that role, and also with my years of experience, I have taken hazardous materials and exposure in confined spaces training.”*

*“[I’ve had] very little safety training. It is ongoing though it is not so much for what you breathe on the job, it’s more for the fact that I have laborers working around me while I’m on an excavator, so there’s more safety training for operating equipment [and preventing injury] around other people than for personal health.”*

*“I’m on the safety committee, and I’ve had training on safety issues, WorkSafe regulations, but nothing that has to do with diesel exhaust.”*

*“I was the safety rep, and I had training about the labor code, though nothing that has to do with diesel exhaust exposure.”*

*“I’ve had training for if something happens and we’re trapped in the tunnels, how to use the respirators. But that’s just for emergencies, to get out of the tunnels, that’s not for exposures. You couldn’t stay inside the tunnel with this type of mask, it doesn’t have a separate canister; it’s just a filter. Other than that, that’s about it.”*

*“I’ve had WHMIS training, though nothing relevant to diesel exhaust.”*

*“No training, [but] because I have been working, I know what makes [the engines] run, what makes [the diesel] smoke blue, why it smokes black, and how to get the right air-fuel mixture.”*

*“We have safety courses, about high voltage, but nothing about exhaust. We just know it’s there.”*

*“Not specific to diesel. I teach occupational safety and health and you have to make sure that you protect yourself as much as possible.”*

**(B) Follow-up probe about reasoning for ranking for diesel exhaust vs. other inhalational hazards.**

*“I would say particles and exposure from fires, and same from diesel. Everyone knows how dangerous smoke is. Those two things are number one.”*

*“I would say paint enamel is a four or a five. That really put me over the edge, that stuff is dangerous.”*

**(C) Follow-up probe about reasoning for agreeing or disagreeing to DE being a carcinogen.**

*“That's common sense, isn't it? Is there any healthy smoke on this planet?”*

*“I do, but again, it's an assumption, it's something that I believe but I don't know why, I can't validate why I think so, but I feel like I have this knowledge that it's carcinogenic.”*

*“I don't think so, no. But I don't know enough, I'm not a doctor. But I don't think so.”*

*“It could be, I don't know. I definitely think it's unhealthy though."*

*“Uh-oh. I hope not! I assume it would, but I don't know that. It can't be good for you. I'm going on the blissful ignorance method here; it might not be the best.”*

**(D) Reasoning for whether they trust or not trust named sources of information (organized by sources of information).**

**WorkSafeBC**

*“WorkSafeBC would be one of the first resources that I’d look to.”*

“*I trust WorkSafeBC but I don’t think they do nearly enough; the people who need it are denied privileges and benefits, and you get the other half who are scamming who don’t have an actual injury who’s milking the system and it costs employers.”*

“*I’m a little skeptical of WorkSafeBC, as I have had dealings with them throughout the years. I’m skeptical to their mandate that is not necessarily in the workers’ best interest.”*

“*WorkSafeBC is well, corrupt. They are going to cover [the employer]’s butt, no matter what.”*

**Internet**

“*I would Google it [on the internet]. But if it were some massive corporation like WorkSafeBC, I would take it with a grain of salt because I don't think they would be posting the truth.”*

*“Well, I would read all the information [on the internet], what they are posting about everything, and if I agree with things that I already know, then I would find that information more credible.”*

*“[…] I would Google [on the internet] the 'health effects of diesel', with all the stuff that would come up, I'd tend to look at stuff that's more familiar to me, or more reasonable, and with links to university projects and things like that.”*

“*Everything that I read on Google [on the internet], I take it as somebody’s opinion, and not written as law.”*

**Doctor**

*“I don’t know where I would go, maybe my family doctor but only if I’m quite ill or something.”*

**Employer or Workplace**

*“I would trust my boss.”*

“*I would refer to material safety data sheets available at my workplace. I would look for toxicity warning on the product cans that had diesel in it.”*

“*Sure, there are bulletin boards at my workplace, and WHMIS for the stuff that’s on the work site, but when it comes to being [a heavy equipment operator], you really don’t see a lot of it, because you’re in an industry where you’re always moving. I might be at one job for two weeks, then I’ll be at another job for two weeks, and so on.”*

“*Surprisingly enough, I would trust the info if it came through the company’s health department, because [in his personal experience, there was another scenario he described], where they would go away or around from any reasonable steps. Since they usually wouldn’t disclose things, if they are to disclose that diesel exhaust does this and that, I would trust them*.”

“*I would say that there are no resources here at [the workplace] because I am not aware of anything offhand, and I’m pretty good at knowing what’s available around here, so if I don’t know easily, then probably others won’t know either. So I would say there’s nothing readily available around here.*”

“*I would not trust [the workplace] because they have other interests other than safety.”*

**Unions**

“*I used to work [at another workplace] where we would have tailgate meetings. These were the workers themselves doing the conversations with the bosses telling the employees to be careful of this and that, and that only happens where you have strong unions.*”

*“From past experience, they just don’t bother, it’s extra work and they just sweep it under the rug.”*

**Health Canada**

“*I might say Health Canada but I’m still not sure if they would have the information.”*

**Health and Safety Committee**

*“[I would] talk to our health and safety committee; they're really good about putting us on documentation, or give us online stuff, or books.”*

“*I don’t trust [the safety committee at the workplace] would have the answers or care to say the truth, because then it would be held against them, which has happened in different situations.*”

**Universities and Research Studies**

“*I want to know where the study is coming from, and I like your study being from UBC (University of British Columbia), and it has to do with the workplace*”.

**Colleagues**

“*Well, the people at my [workplace], yeah [I would trust them]*.”

“*I was [wearing a mask], and people make smart-ass remarks, from all the [colleagues], there’s no help from them.”*

**Government**

“*[I would trust] anything that’s regulated by the government.”*

“*I would look for any sort of studies that were government-issued, and what I would look for is something that’s not of private interest. I’d like to see something where someone is giving me information as opposed to giving me an opinion.”*

“*I probably won’t trust any government agencies; I tend to take with a grain of salt. Not-for-profit agencies, I tend to have a bit more faith in because they have no reason to skew things one way or another.”*

**(E) “What actions do you take at your workplace to address diesel exhaust exposure? Please name as many as you could” (organized by categories of hazard control).**

**Personal Protective Equipment**

*“I did buy a mask or a respirator. And I used it for a while. And then I asked the safety and training rep, 'When is the company going to purchase masks for us?', and he just laughed. He said 'It's not going to happen'. It’s too expensive to buy on our own. I don't wear it anymore. It's kind of a pain in the neck. And people make smart-ass remarks, from all the other drivers. I stuck with it for a few weeks. I only use it when it's really cold out, zero to two degrees, or below zero, then I'll wear it.”*

*“What could I do [when responding about the actions that he takes to address being exposed to diesel exhaust at his workplace]? I was wearing a respirator when I was painting, but other than keeping ventilation fans going, there was not much that I could do.”*

“*Some people react to it more than others, so if one person reacts to it, then he should wear a filter mask.”*

**Administrative Controls**

“*Many [of us] will go and do [a part of our job] quickly and it’s getting more rigorous and there’s more paperwork, so if we miss something in [a part of our job] and there’s an accident because we didn’t want to smell the diesel fumes, the law will come down onto us.”*

“*Geez, I can't think of anything [else]. I hold my breath when a big cloud is coming towards me, but that probably doesn't help.”*

*“[I’d] hold my breath for as long as I can. I'll put a cloth or part of my shirt over my nose and mouth.”*

“*If I see a situation where there's a lot of diesel smoke, I try to avoid it, like holding my breath or turning to another direction.”*

“*I’ve only documented when I went into the hospital. Or else I’ll be filling out one every shift. Which is probably what it would take, for it to be recognized when it comes time of retirement. All of a sudden, your lungs are not working.”*

*“[I’ve filled out the form] usually for smoke inhalation, or chemical exposure, or diesel, or unknown gases. The form asks about the kind of exposure, the duration, the kind of protective equipment you have on at the time, what did you do about it, what kind of medical attention was necessary, did you seek medical attention, and there are a couple of other things on there that I can’t remember. We submit them to our health and safety committee. If we feel ill, then it becomes a workplace accident. If you seek medical attention, then it's a WCB thing. And these submissions follow you throughout your career. So at the end of your career, you can bring up your exposure list, and it'll tell you how many times you've recorded an exposure.”*

*“Some of the [other people] that I work with, they have monitors and they carry them on the waist of their belts, and if the exhaust gets really bad where they're working, because we do a lot of work besides traffic, and if it gets really bad, there's no wind and it's stale, the alarms go and we put on a mask, not quite a respirator, I have used respirators before, but most of the time it's just a mask, or you close your door or windows.”*

“*Whenever I'm [at work], I have my own carbon monoxide tester unit up on the dash with me, it has never ever gone off, so I don't know [if the level has been high enough for me to get sick as well]. [A colleague] and I got our own units, little battery-operated units, and I went to the company and said you know what? It only costs me $29.95, why doesn't the company just get everyone one, and they said no, because they might hold it down by the exhaust pipe and make it go off and say that the [vehicle] is no good.”*

“*We try to shut down the engines as soon as possible, and as much as possible.”*

“*We’ve done public relations events, and instead of just having me do it myself, we had people from environmental consultant side, and there were displays up talking about the effects of the diesel exhaust on the human body, and we also had up the numbers of how often the [vehicles] are sitting idling which we look at quarterly, and also have it on a bulletin board. And we also put stickers on the ignition switch, ‘help clear the air, and reduce idling’ to put the thought into the guys’ heads.”*

*“We’ll have tailgate meetings to remind [colleagues], like ‘don’t let the [vehicles] idle, if it doesn’t have to be on, then shut it off’”*

**Engineering Controls**

“*Mechanically it’s getting the diesel exhaust burned more efficiently, then you’re not breathing in raw fuel.”*

“*Ventilation is key. They have exhaust systems, that’s all good. They’re pushing in cleaner, fresher atmospheric air. I’m in Prince George with two pulp mills, and they tell you that the air quality is so poor to stay in the house. But that’s hard to do when you have to go to the shop with the diesel fumes.”*

“*Two or three of us made fiberglass covers in the [area where the diesel exhaust is] to avert the fumes. And it actually did make [the situation with the diesel fumes] better.*”

*“That is natural ventilation, with the large doors opening, we’d have cross-ventilation.”*

*“The new and modern excavators, they have internal and external filters for the cab, or the part that you sit in. So keep your windows and doors closed. And use the ventilation system, and you have the ability to choose from drawing the air from within the cab, or from the exterior. If you're smart, you can really control the diesel fumes in that respect.”*

*“If I'm next to a vehicle that's spewing exhaust, then I would close my window. Oh, also there's a vent at our feet at the front of the bus, [which] opens up in the summer. You can open it anytime that you want. It's right at your toes, it lets cold air in, and it comes up your feet. I never, never, never, ever, open that. I always close it. Because it's right at the height of the tail pipe. And a lot of other people open that vent and I can’t believe it!”*

**No Action**

*“Since [they are] not high levels, I don’t really do much. I don’t wear masks. I just continue like nothing is going on. I just smell it.”*

**(F) “Share your recommendations for how we can better address the exposure of diesel exhaust at your workplace, whether it was by yourself, fellow workers, the workplace, the government, etc. Name as many recommendations as you can think of” (organized by categories of hazard control).**

**Personal Protective Equipment**

“[*The workplace] can provide proper protective equipment and training and let people know the actual hazards, potential hazards, basically give them all the tools to make the right decisions.”*

“*What we’ve been given are not suitable. I’m sure there are other ones in the market that could work, but once again, it comes with a price. The workplace is not going to supply it, that’s the biggest thing, the expense. Even if there’s a regulation for the workplaces to provide the masks, I think they can come up with a better system to ventilating the exhaust out, there’s got to be better ways.* ”

“*It’s not realistic to think that all workers will be wearing masks, we won’t. We have to talk a lot on two-way radios. So it can be dangerous if we’re not audible and heard clearly. Masks can be hot. It’s just impractical.”*

“*[Workers] are still eating and breathing [the diesel exhaust] because there’s no way around it, they can’t wear masks because it’s cumbersome and restricting, I don’t think the boss would go for it. Or the next one is the little gas masks, look at the Chinese and Japanese, they wear it daily from work, to work, to try to get a little bit more life on this planet. We're not quite that bad at the workplace, but we can still have an improved filtration.”*

“*People need to be reminded that it is not optional to use [the protective equipment], your employers are telling you to use it, and the expectation is that you use it.”*

**Administrative Controls**

“*The workplace can better educate their employees, with our bad habits, when I was younger, it’s because I wasn’t aware of the long-term health effects, but if we had proper education on exposure and how to avoid exposure, I wouldn’t have allowed myself to get into some situations that I got myself into.*”

“*More education would be good, so more people would hold their breath [when they are near the diesel exhaust]. I know I would hold my breath. Maybe with more education, because people are so sick of bulletins!”*

*“Unfortunately if someone dies from something, maybe capitalizing from that, we really need to expose that, like why they died, and what they died from, and education people like ‘look, this person could be healthier and have a healthier life if they weren’t exposed to this or did everything they could to avoid it.’”*

“*I think there can be more [formalized education] when someone is hired. There should be more awareness made that [diesel exhaust] is one of the things that you're dealing with at this workplace. And this is what you could do to help prevent, [for example] wearing masks, finding a different shift time, or [picking up the diesel vehicle somewhere else]. If the exhaust is causing you discomfort, these are things that you can do.”*

*“It would be great to give more information to employees who are affected, give links about studies that have been done on it. It needs to be taken more seriously. For years for me, I just put up with it. I suppose info [should go] to anyone who works with anything that emits exhaust, like diesel, gasoline, etc.”*

“*I don't know if the technology is there yet, but if they can have some sort of sensor by where the workers work the vast majority of the shift, and they can see what percentage they're inhaling per shift*.”

“*Well [the workplace] can definitely come down here for a visit and bring the air sniffers and measure the levels. In the 35 years that I've worked here, I've only seen people come here twice, once to measure sound levels, and the other time it looks like it was for the fumes, it was around 25-30 years ago, they walked around with meters, but they didn't do anything else, and we never saw a report. There was probably a report to the higher-ups, but we were never notified of anything. I'd like to see them spend more money on studies and doing measurements.*”

*“Let's say if a company knows that their employees are exposed, then they should measure the levels [of diesel exhaust], and the policy makers can say if it's this level or above, then they have to wear a mask.”*

*“I'm not too sure what is the daily limit in Vancouver, maybe 20ug/cubic meter on a daily basis then they have to wear a mask.”*

“*Workers' Compensation Board or WorkSafeBC has a yearly or every two years hearing test requirement so that if you have hearing problems later on in life, they would have a record that you've been tested 30 times in 30 years, and your hearing has slowly gotten worse, even though you have worn the industry hearing plugs. Now they should do the same for diesel so you could test the person's blood or urine or lung capacity, I'm not sure how they actually test the lungs, but I think that's a recommendation in regards to going to the next step. If this is a pilot project, maybe that's where this should go, because [stuff] in the diesel exhaust affects the human body the same way everything else does, so maybe this is something to look at.*”

“*I know no employer would do it, but they should test people when they hire people on, like lung capacity, to get a baseline. There are other factors too, like if you smoke, but if you have a baseline, then you can tell if there's a problem later on. With people like me, we don't have a baseline to say, this guy was perfectly healthy when they were hired on and now he has this asthma problem that we think has to do with diesel exhaust.”*

*“[Either the workplace itself] or health researchers can go to the workplace to survey the employees, specifically one-by-one, what are your thoughts, your feelings, how are you doing, have you had any lung problems, any respiratory issues, and essentially research enough people so that you can get a straight answer.”*

“*My company can actively go after all employees who are leaving vehicles idling unnecessarily. In other words, get the managers more involved, rather than just turning a blind eye. Big companies, it's hard and slow to change opinions and attitudes but we need to do that. We need to get people realizing that newer vehicles don't take a long time to warm up. [...] So the company can do more PR, and also go after people. Everyone wears seatbelts now, but in the 60's, you didn't. You also don't smoke in restaurants and hotels anymore. You have to swing the pendulum and push harder. You have to start somewhere and push it. We're not giving enough pressure.”*

“*[There are] different measures that you can probably use, both facility-wise and procedure-wise to minimize potential exposure.*”

*“[In situations with an extreme amount of diesel], there can be some sort of regulation, like stand back 50 feet, or something, I don’t know if that’s enough to make a difference.”*

“*My company can actively go after all employees who are leaving vehicles idling unnecessarily. In other words, get the managers more involved, rather than just turning a blind eye. Big companies, it's hard and slow to change opinions and attitudes but we need to do that. We need to get people realizing that newer vehicles don't take a long time to warm up. [...] So the company can do more PR, and also go after people. Everyone wears seatbelts now, but in the 60's, you didn't. You also don't smoke in restaurants and hotels anymore. You have to swing the pendulum and push harder. You have to start somewhere and push it. We're not giving enough pressure.”*

“*I’d say that we can’t really limit the exposure but we could remove ourselves from the environment.*”

**Engineering Controls**

“*There would be ventilation for each [area section], that would essentially bring fresh air from outside, process the diesel exhaust and put it on the other side of the [area where workers work at].”*

“*Be abit more aggressive with tactics to remove it from enclosed areas, proper ventilation to minimize exposure, tougher regulations*.”

“*The government needs to crack down on emissions. The time frame or the allowed threshold is too generous. You have five or 10 or 15 years to cut down on your emissions to an acceptable level. They gave corporations 20 years to bring it down. I might not even be here in 20 years! I think it should be a short period of time, get it down, or you get fined. Same with these trucks, these big four-wheel drives, with black smoke. That's how you get black smoke with diesel, the filters are clogged down. There's a penalty, here's a ticket, get your truck maintained.”*

*“I believe the provincial or the federal government, and I am not holding my breath on this one, should do regular inspections on all heavy equipment, heavy diesel engines, doesn't matter if it's a pick-up truck, a boat, a tractor-trailer, a bulldozer, a loader, or an excavator. Every piece of diesel equipment on planet earth, should be inspected by a federal inspector, can be passed off to provincial, whatever, that's fine with me. But I do not see an inspection that is working for diesel exhaust. But only if you have license plates do you have to get your dump truck checked. But I don't think they check diesel exhaust. It's very unregulated. It needs to be as regulated as the automotive industry is in regards to making the gasoline engines run, not so much in terms of efficiency, but in environment sensitivity.”*

*“Ideally, it would be great if we can go to electric vehicles, with less particles. I know [my workplace] has some, but the majority are diesel.”*

*“Maybe the policy makers can regulate the diesel exhaust a little better. I know that the new pick-up trucks, the exhaust is way better. You don't notice it as much, but they have that for pick-up trucks but not for trains.”*

“*the government could put in place a law that requires all diesel engines to be converted to be able to run the ultra-low-sulfur diesel , and if not the engine can be treated with a catalytic converter so that the exhaust can be as clean as possible. I say that because I believe that the government will be requiring vehicles to have certain inspection criteria. That will be a good situation. I want to see something being done with the kind of fuel that they're burning, or how it's burned. It seems that that's a big source of the smog and particular matter in the air.”*

“*More maintenance, more vigilance with checking with [equipment that contains the diesel exhaust], possibly that can help down the road.”*

*“[I’d like to see them] continue with improvements, facility improvements, make sure ventilation systems are incorporated into the building, I think new vehicles are more efficient and less polluting than the old equipment, they start better, produce less emissions, so I guess to just keep up with continued improvements.”*

**No Recommendations**

“*I honestly don't know, because of the nature of the beast, [referring to the exposure of diesel exhaust]. Those engines have to run to do what they have to do.*”

“*I don't know other than being aware of it and cleaning it up a little bit, but it's the most efficient fuel that we have, and that's all people care about, how much money they can save. So I don't see it changing a huge amount, until people get sick, and it costs them more to keep them healthy than it does to get rid of the diesel. That's what I see*.”

“*I think they're doing a great job already, and they're pretty strict on policies that they've put in place regarding [diesel exhaust]. And they do a good job following up whenever they hear of people slacking off a bit. They put in notices to remind people. So no, I think it's as good as it gets.*”
